# Thalamic Ganglioglioma Treated With Radical Radiotherapy: A Rare Location and an Exclusive Form of Treatment

**DOI:** 10.7759/cureus.72171

**Published:** 2024-10-22

**Authors:** Luísa Samarão, Artur Aguiar, Manuel Jacome, Madalena Souto de Moura, Mavilde Arantes

**Affiliations:** 1 Radiation Oncology, Portuguese Oncology Institute of Porto, Porto, PRT; 2 Pathological Anatomy, Portuguese Oncology Institute of Porto, Porto, PRT; 3 Neuroradiology, Portuguese Oncology Institute of Porto, Porto, PRT; 4 Centro de Investigação do IPO Porto, Portuguese Oncology Institute of Porto, Porto, PRT

**Keywords:** brain tumor, ganglioglioma, neuronavigation guided brain biopsy, radical external beam radiotherapy, thalamus

## Abstract

Gangliogliomas (GG) are rare primary central nervous system (CNS) tumors. These CNS tumors are more commonly located at the supratentorial level. The treatment of choice for these tumors is surgical resection, and the role of radiotherapy remains controversial. A 61-year-old woman who presented with seizures underwent a magnetic resonance imaging (MRI), which showed a left-side thalamic lesion with one solid and two cystic components. A neuronavigation-guided brain biopsy of the lesion established the diagnosis of GG, with expression of glial fibrillary acidic protein (GFAP) for glial cells and neuron-specific enolase (NSE) and synaptophysin for ganglion cells. Due to the location of the lesion, the patient underwent radical radiotherapy. Post-treatment MRIs revealed a reduction in tumor dimensions. In conclusion, we emphasize the role of radiotherapy in the treatment of cerebral GG.

## Introduction

Gangliogliomas (GG), histologically, are a mixture of well-differentiated neoplastic ganglion and glial cells [1]. According to the World Health Organization (WHO) grading, the majority are indolent and designated as grade 1 tumors in the current classification of central nervous system (CNS) tumors, although in a minority of cases, higher grade features are present [2].

GG are tumors most frequently diagnosed in children and young adults presenting with seizures [3]. GG can be localized in any part of the CNS, although they are more commonly supratentorial and predominantly within the temporal and frontal lobes [4]. According to a review of literature, this tumor is rare with very few clinical cases located in the thalamus [5].

The treatment of choice for these tumors remains surgical resection. Radiotherapy (RT) is usually reserved for progressive or malignant tumors after surgical treatment. Tumor resection might not always be feasible, due to possible deep brain location and vicinity to critical brain areas, and these cases require alternative or additional treatments. To the best of our knowledge, the effect of RT on GG as a first-line treatment has not been investigated.

In this case report, we describe a 61-year-old woman diagnosed with a GG located in the left thalamus and treated at our institution. This article was previously presented as a meeting abstract at the 2023 Oncology Spring Meeting, held in Portugal from the 23rd to the 25th of March, 2023.

## Case presentation

The patient, a 61-year-old woman, was admitted to our institution with seizures. On the neurological examination, there was no specific sign. With regard to medical and surgical history, she had hypertension, obesity, and depression and was previously submitted to tubal ligation and phlebectomy due to venous insufficiency. Medication included lisinopril with hydrochlorothiazide, fluoxetine, lorazepam, and bupropion.

**Figure 1:**
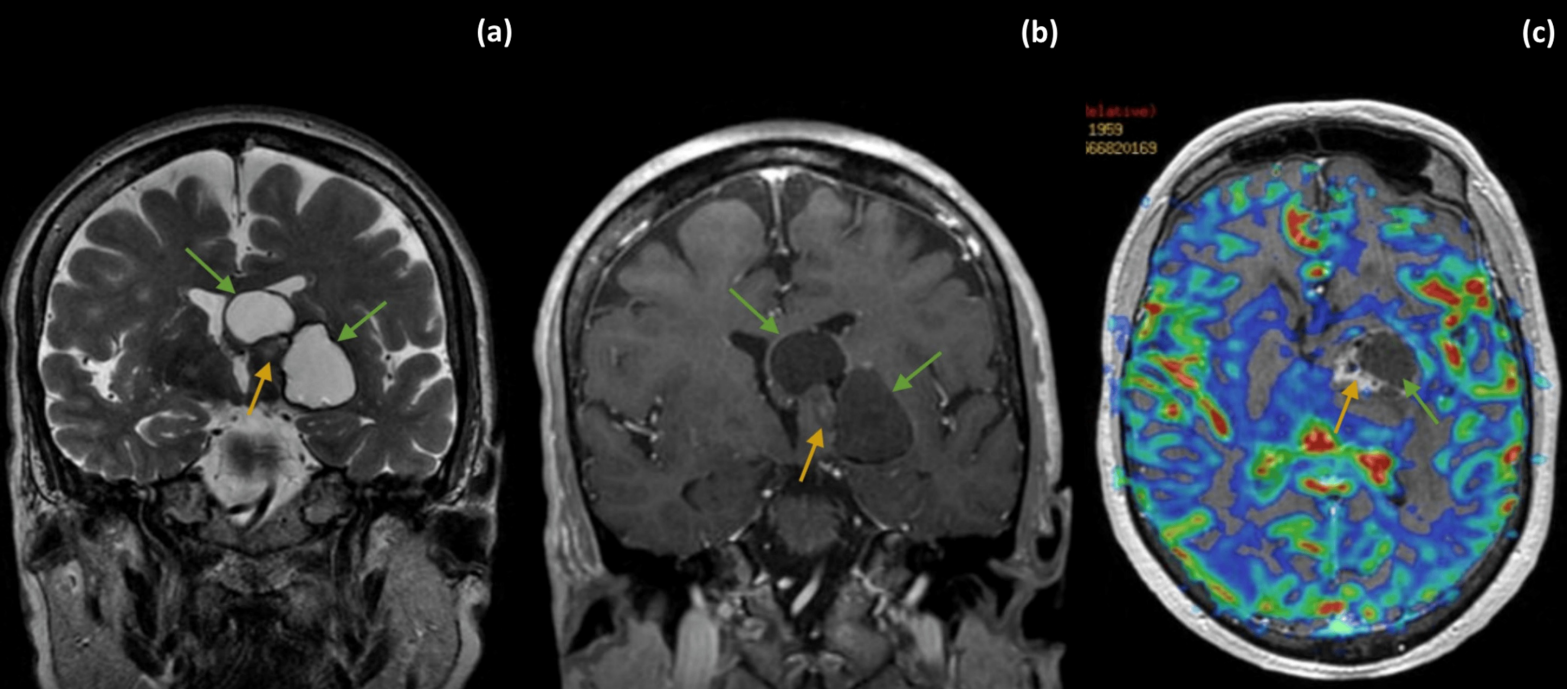
Diagnostic brain MRI. Brain MRI showed a left-side thalamic lesion with one solid and two large cystic components, one extending on the left lenticulocapsular region and the other encroaching to the body of the left lateral ventricle. The two cystic components (green arrows) were homogeneously hyperintense on T2-WI (a), with no contrast enhancement on post-contrast T1-WI (b). The solid component (yellow arrow) of the lesion showed mild heterogeneous enhancement on post-contrast T1-WI (b). On T2-WI (a), there is also visible discrete vasogenic edema in the parenchyma surrounding the lesion and right midline shift of the brain at the level of the septum pellucidum. There is no apparent increase in perfusion on the rCBV map (c). rCBV: relative cerebral blood volume

A computed tomography (CT) of the brain revealed a left-side thalamic lesion with one solid and two large cystic components (one extending on the left lenticulocapsular region and the other to the body of the left lateral ventricle). On magnetic resonance imaging (MRI) the two cystic components were homogeneously hyperintense on T2-weighted images (T2-WI) and hypointense on T1-weighted images (T1-WI) with no contrast enhancement on post-contrast T1-WI. The solid component of the lesion showed mild heterogeneous enhancement on post-contrast T1-WI. The tumor caused mass effect with surrounding vasogenic edema, subfalcine herniation, and rightward midline shift in about 0.8 cm. There was no apparent increase in perfusion on the relative cerebral blood volume (rCBV) map (Figure 1).

**Figure 2:**
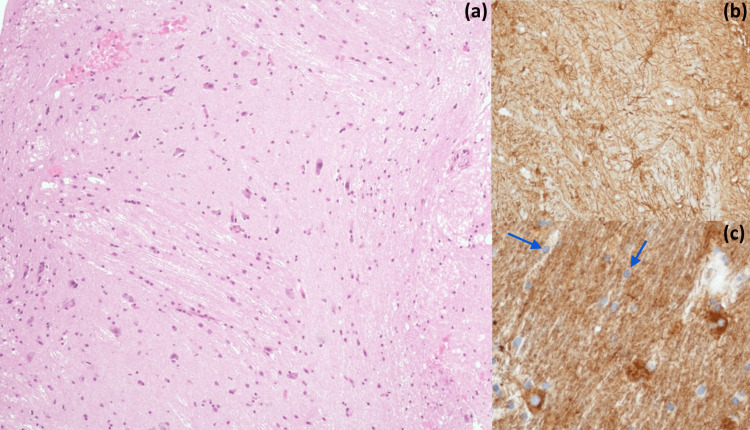
Histopathologic and immunohistochemical examination of brain biopsy. Ganglioglioma (a) with a fibrillary glial component with minimal atypia and positive for GFAP (b) and scattered admixed atypical neuron-like cells, highlighted by synaptophysin (blue arrows) (c). GFAP: glial fibrillary acidic protein

The patient underwent a neuronavigation-guided brain biopsy of the lesion. Histopathological examination revealed morphological and immunohistochemical aspects suggestive of well-differentiated neoplasia consisting of both a ganglionic and glial component (Figure 2). Glial cells were positive for glial fibrillary acidic protein (GFAP) and ganglion cells were positive for neuron-specific enolase (NSE) and synaptophysin. Ki-67 labeling was < 1%. The sample did not show the oncogenic variants of the BRAF gene. Thus, the observed aspects were consistent with a diagnosis of GG, classified as WHO grade 1.

**Figure 3:**
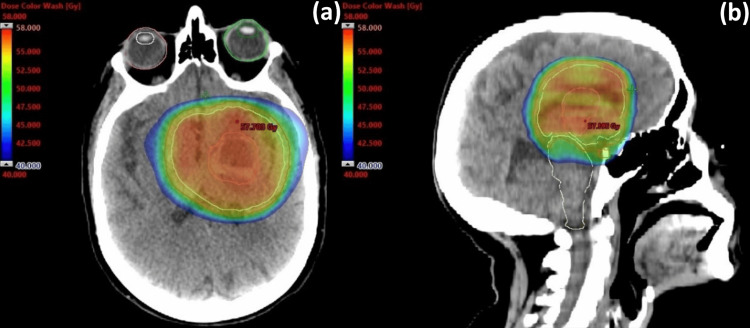
RT dose distribution to tumor. Isodose distribution curves of radiotherapy, in axial (a) and sagittal (b) views. RT: Radiotherapy

In the face of these findings and due to the fact that the tumor was considered unresectable given its location, a multidisciplinary team proposed treatment with curative-intent RT. RT was performed with Volumetric Modulated Arc Therapy (VMAT) for a total dose of 54 Gy, delivered in 27 fractions of 2 Gy per day, five times a week. The gross tumor volume (GTV) was delineated based on planning CT and MRI, then a 1.5 cm expansion was made to the clinical target volume (CTV), which was limited to anatomic barriers. The planning tumor volume (PTV) was obtained using a 0.3 cm margin expansion from the CTV (Figure 3).

**Figure 4:**
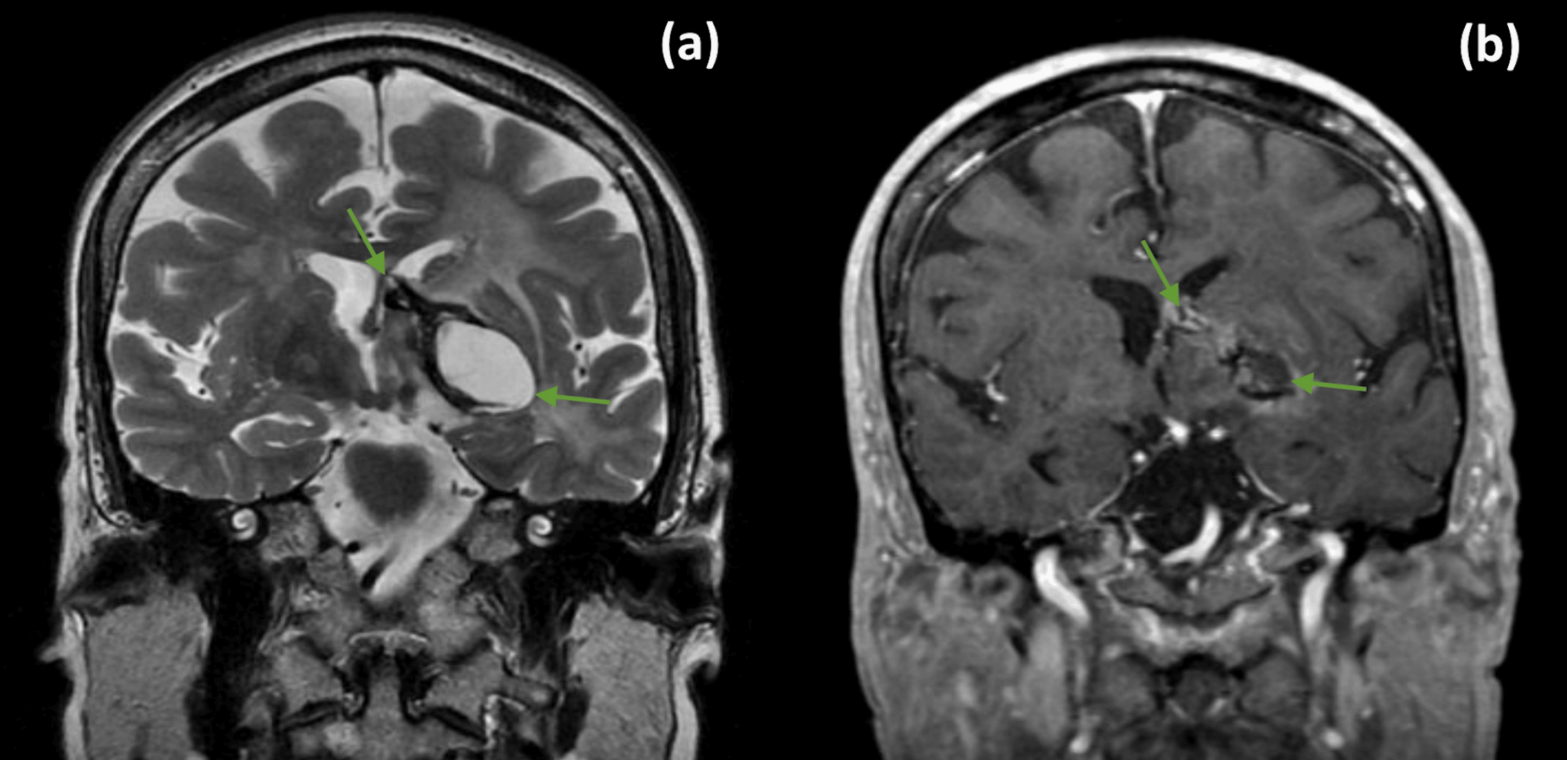
Brain MRI 6 months after RT treatment. T2-WI (a) and post-contrast T1-WI (b) revealed a reduction in tumor dimensions, particularly of the cystic component (green arrows) that extends to the body of the left lateral ventricle.

She was treated for 40 days, with no interruptions experiencing only grade 1 nausea throughout the treatment sessions. Post-treatment MRI (six months after RT) revealed a reduction in tumor dimensions (Figure 4).

The patient is regularly followed to monitor disease status and treatment-related adverse events. Four years later she is alive with a stable disease.

The presented case is remarkable since it regards a thalamic GG, in an adult woman, treated exclusively with radical RT with a favorable outcome. Although it occurs rarely, GG should be included in the differential diagnosis of a thalamic mass because early recognition is important for treatment and patient counseling.

## Discussion

GG are low-grade glio-neuronal neoplasms that account for 0.3-1.4% of all CNS tumors [6,7], with the temporal lobe being the most common location (>70%) [8,9]. They are also seen in the frontal, parietal, and occipital lobes, along with the brainstem, spinal cord, optic chiasm, and pineal region. Thalamic GG are rare, with very few cases, including ours, described in the literature [5,10, 11].

On average, the age at diagnosis of brain GG is between 16 and 29 years of age [12]. GG represents <1% of primary brain tumors in adults [13]. While GG in children and young adults has an excellent prognosis, older age could indicate a worse prognosis. Therefore, the presentation at 61 years of age and having stable disease after 4 years in our report are rather rare occurrences.

The most common presentation of brain GG is seizures, which are reported to be long-standing and drug-resistant, with an incidence of 75-100% in the literature [14]. Through literature review, cases with GG specifically located in the thalamus have different clinical presentations [5,10, 11]: one of them presented with hemiparesis and paresthesias in the limbs; other cases presented with hand tremor, and one of them had a defect in the visual fields. In our case, seizures were the only presenting symptom.

Regarding imaging, GG commonly presents with solid and cystic components. On MRI the cystic component may show isointensity to hypointensity on T1 compared to the gray matter, and isointensity or hyperintensity on T2 compared to the normal parenchyma [15], as in our case.

The histopathologic features of GGs are characterized by a moderate, inhomogeneous cellularity with a marked cytonuclear pleomorphism [16]. Histologically, GFAP staining highlights the glial components, whereas positive staining of synaptophysin, chromogranin-A, and NeuN can label the neuronal/ganglion cell components in the GGs [17]. Moreover, immunohistochemistry for BRAF V600E, which is a common molecular feature of GGs, may also be helpful [18]. GFAP, NSE, and synaptophysin were positive and BRAF mutation was negative in the previously reported case.

The first treatment option for GG is complete surgical resection whenever possible and the role of RT remains controversial. Although the importance of RT in cases of subtotal resection and in recurrences that are unsuitable for further surgical resection are already documented, the value of radical RT as the first treatment option has never been reported. Rades et al. [19] in a retrospective study of 402 patients with GG compared four different treatment regimens: gross total resection alone (GTR), GTR plus RT, subtotal resection alone (STR), and STR plus RT. In relation to the surgery groups, overall survival (OS) and local control (LC) were significantly better in the GTR group than in the STR group. RT combined with surgery did not significantly improve OS in any of the subgroups. However, LC was significantly improved with the addition of RT in low-grade and high-grade GG. Regarding the treatment of thalamic GG cases described in the literature, one case was treated with surgery alone (trans-Sylvian approach) [11] and the other two cases were treated with surgery and postoperative RT [5,10].

To the best of our knowledge, this is the only described case of a thalamic GG treated exclusively with RT.

## Conclusions

In conclusion, although thalamic GG is a very rare entity, it must be considered in the differential diagnosis of brain tumors presenting with seizures, even in middle-aged adults. A correct diagnosis by morphology and immunohistochemistry is of vital importance to avoid non-beneficial treatments.

This case allows us to reflect on the different therapeutic modalities in inoperable GG, highlighting the role of radical RT in the treatment of these tumors. Further studies are needed to clarify the efficacy of radical RT in this context.
